# Dairy product consumption, eating habits, sedentary behaviour and physical activity association with bone mineral density among adolescent boys: a cross-sectional observational study

**DOI:** 10.1186/s12887-024-04539-y

**Published:** 2024-01-17

**Authors:** Anna Kopiczko, Michał Czapla, Raúl Juárez-Vela, Catherine Ross, Bartosz Uchmanowicz

**Affiliations:** 1 Department of Human Biology, Józef Piłsudski University of Physical Education in Warsaw, Warsaw, Poland; 2 Department of Emergency Medical Service, Wrocław Medical University, Wrocław, Poland; 3 Institute of Heart Diseases, University Hospital, Wroclaw, 50-566 Poland; 4 Group of Research in Care (GRUPAC), Faculty of Health Science, University of La Rioja, Logrono, 26006 Spain; 5 Centre for Cardiovascular Health, School of Health and Social Care, Edinburgh Napier University, Edinburgh, EH11 4BN UK; 6 Department of Nursing and Obstetrics, Faculty of Health Sciences, Wroclaw Medical University, Wroclaw, Poland

**Keywords:** Bone health, Adolescents, Physical activity, Sedentary behaviour, Eating habits, Dairy products, Calcium, Protein, Vitamin D, Phosphorus

## Abstract

**Background:**

During childhood and adolescence, skeletal microarchitecture and bone mineral density (BMD) undergo significant changes. Peak bone mass is built and its level significantly affects the condition of bones in later years of life. Understanding the modifiable factors that improve bone parameters at an early age is necessary to early prevent osteoporosis. To identify these modifiable factors we analysed the relationship between dairy product consumption, eating habits, sedentary behaviour, and level of physical activity with BMD in 115 young boys (14–17 years).

**Methods:**

Bone parameters were measured by dual energy x-ray absorptiometry using paediatric specific software to compile the data. Dairy product consumption and eating habits were assessed by means of a dietary interview. Sedentary behaviour and physical activity was assessed in a face-to-face interview conducted using the International Physical Activity Questionnaire. Data collection on total physical activity level was performed by collecting information on the number of days and the duration of vigorous and moderate intensity (MVPA) and average daily time spent in sitting (SIT time).

**Results:**

The strongest relationships with BMD in distal part of forearm were found for moderate plus vigorous activity, sit time, and intake of dairy products, intake of calcium, protein, vitamin D, phosphorus from diet. Relationships between BMD, bone mineral content (BMC) in the distal and proximal part of the forearm and PA, sit time and eating parameters were evaluated using the multiple forward stepwise regression. The presented model explained 48–67% (adjusted *R*^2^ = 0.48–0.67; *p* < 0.001) of the variance in bone parameters. The predictor of interactions of three variables: protein intake (g/person/day), vitamin D intake (µg/day) and phosphorus intake (mg/day) was significant for BMD dis (adjusted *R*^2^ = 0.59; *p* < 0.001). The predictor of interactions of two variables: SIT time (h/day) and dairy products (n/day) was significant for BMD prox (adjusted *R*^2^ = 0.48; *p* < 0.001). Furthermore, the predictor of interactions dairy products (n/day), protein intake (g/person/day) and phosphorus intake (mg/day) was significant for BMC prox and dis (adjusted *R*^2^ = 0.63–0.67; *p* < 0.001).

**Conclusions:**

High physical activity and optimal eating habits especially adequate intake of important dietary components for bone health such as calcium, protein, vitamin D and phosphorus affect the mineralization of forearm bones.

## Introduction

Bone tissue is the basic element of the skeleton, having a mainly supportive function, it is also involved in the protection of the organs. The most important quantitative bone parameters are subject to constant change during the different periods of ontogenesis. There are three main processes regarding bone growth: bone formation, bone modeling, and bone remodeling (BR). The main task of remodeling is the protective function of the structure of the skeletal system as well as ensuring the mineral balance of bone tissue [1, 2].

Bone remodelling occurs at very different rates in the adult life. During periods of dynamic physical development, i.e. during childhood and adolescence, skeletal microarchitecture, bone mineral content (BMC) and bone mineral density (BMD) undergo significant changes. The process of growing BMC and BMD in the early years of life is a major determinant of bone status later in life. Once skeletal maturity is achieved, BMC is defined as peak bone mass (PBM). Many factors influence the construction of PBM. Research indicates that about half of PBM is acquired during adolescent years, with complete PBM reached around 30 years of age. Importantly, PBM which is too low is the predictor of osteoporosis and increased fractures in the later years of life [3, 4].

Studies have shown that genetics plays an important role in determining bone parameters [5]. However, studies have shown that environmental factors such as eating habits, dietary pattern [6, 7], type of diets especially the intake of calcium, vitamin D and protein, level and the type of physical activity (PA) [8, 9] can be important factors in determining bone health. The findings also underscore the importance of the synergistic interaction of protein intake and exercise in the early prevention of low PBM and the development of osteopenia risk in the adolescent population [10]. The World Health Organization (WHO) recommends both PA and calcium intake to maximize PBM in children and adolescents [11]. Children's health studies are now paying particular attention to the effects of sedentary lifestyles on bone status [12–14]. During the pubertal stage, bone parameters change at a significant rate, and by the end of puberty, bone mass reaches almost the full PBM characteristic of an adult [15]. Findings suggest that PBM is not achieved across the skeleton at the same rate, Wren et al. in 2007 suggest that PBM in the lumbar spine has optimal values already at the end of sexual maturation [16], while others demonstrated that bone mineral accrual and PBM at the spine occurs around age 30 years or later. Another study showed hip PBM has been noted to occur between age 16 and 19 years in women [4]. Bone mineral density of children and adolescents is also related to hand strength levels [17]. Achieving optimal PBM during childhood and adolescence is important in reducing the risk of developing osteoporosis later in life. There is a need for new research to better understand what health and anti-health behaviours affect childrens health, and BMD. According to research, body composition and health behaviour are influenced by socio-economic factors [18–20]. Among the most important factors isthe level of PA [21, 22].

The aim of this study was to analyse the relationship of dairy product consumption, eating habits, sedentary behaviour, and level of physical activity especially moderate to vigorous (MVPA) on forearm BMD in two points distal and proximal in 115 young Polish boys aged 14–17 years. This study tested, and hypothesised that high MVPA and optimal eating habits, protein intake, minerals and vitamin D in diet would be positively associated with forearm BMD and BMC and offset the effects of high SIT time. Implications from the verification of the hypothesis may be significant for better defining modifiable factors affecting bone status in young adulthood, which may be useful for effective early prevention of osteoporosis. It is particularly important to verify whether sedentary time, common in the lifestyle of today's adolescent population, significantly affects low BMD in young boys.

## Methods

### Sample and study design

This cross-sectional observational study involved 115 Polish boys aged 16.05 ± 1.0 years. All boys were of the same ethnic origin (Caucasians of European origin). The sample was drawn from the Warsaw Population Screening Study, which is an ongoing, cross-sectional observational bone health. The sample size was calculated using the formula Gibson et al. [23], taking into account the individual variability in BMD and the standard error, taken from published data of the same population, from the same region of Poland [24].

We have used a deliberate random model of group selection, as we have intentionally selected a specific age range and gender, The invitation for participation in the study was sent to randomly chosen schools from Warsaw city (Bielany, Żoliborz, Bemowo districts). After verification of the inclusion and exclusion criteria with the parent and the boy, measurements were taken of those boys who qualified for participation. A total of 312 boys applied to participate from that cohort 97 were excluded, The reasons for exclusion from the study wereboys who did not join the full study or who resigned during the project. All young boys included in the study, and their parents were informed of the aims and schedule of the study. The final cohort consisted of boys who were invited to participate, and did not have the diseases described in the exclusion criteria. The exclusion criteria included bone metabolic diseases, kidney disease, thyroid and parathyroid diseases, cancers, rheumatoid arthritis, and long-term steroid treatment. The study included boys who, according to their mother’s interview, were assessed as healthy full-term newborns (i.e., born between 38th and 42nd week of pregnancy).

Pubertal age was established with percentile curves of stature and weight according to chronological age and growth peak [25]. The studied group of boys was characterised by a normal, average rate of puberty. The project was carried out from October to December 2022 in the densitometry and anthropometry laboratory in the Department of Human Biology at the Józef Piłsudski University of Physical Education in Warsaw. The team of qualitified anthropologists with the appropriate experience in research performed the measurements on the entire study group.

The study was approved by the Bioethics Committee of the National Institute of Public Health, National Institute of Hygiene in Warsaw (protocol number 1/2021) and conformed to the Declaration of Helsinki. Written and informed parental consent was obtained for each boys participation, as well as participant assent obtained for this research project. STROBE (Strengthening the Reporting of Observational Studies in Epidemiology) guidelines were followed.

### Bone tissue parameters, anthropometric measurements

Bone parameters of the forearm in distal (dis) and proximal (prox) locations were measured by means of dual-energy X-ray absorptiometry. Norland Bone Densitometer (Swissray-USA, Norland Medical Systems Madison WI, USA) model Stratec was used, with the effective dose (μSv) of 0.05. Peadiatric software available on this model of densitometer instrument was used. The length of the non-dominant forearm was measured using large anthropometry calipers (GPM large spreading caliper, Zurich, Switzerland). Using the Norland apparatus, measurements were taken in the general distal site and 1/3 proximal site on the radius and ulna. The data analysis was based on bone mineral density (BMD in g/cm^2^), bone mineral content (BMC in g), T-scores, Z-scores and % age-matched. All measurements were taken and analysed by the same individual who is qualified in performing peadiatric measurements. Daily quality control and calibration of the equipment was performed. The coefficient of variation was not determined as it was considered unethical to measure a child several times [22]. Somatic measurements were taken as recommended to the standards of the International Society for the Advancement of Kinanthropometry [26]. A JAWON MEDICAL X-SCAN PLUS II analyzer (Certificate No. EC0197 for medical devices) was used. Body height was measured to the nearest 0.1 cm using the Martin Anthropometer (GMP, Switzerland). Body mass index (BMI) was calculated in accordance with the methodology recommended by WHO [27]. Kinanthropometric measurements [28] were taken by an experienced Anthropologist with qualifications in somatic measurements of children.

### Eating habits evaluation method

Eating habits were assessed using a standardized dietary assessment questionnaires in a face-to-face interview with the parents of the boys and boys. The food frequency questionnaire (FFQ) was used to assess the frequency of dairy product consumption for the three months preceding the survey. Food frequency questionnaires (FFQs) are usually self-administered, in this study after self-administeration a follow-up interview was conducted with the respondent to verify the accuracy of the nutrition data. In addition to the food list and the consumption frequency category section (non-quantitative FFQs), the FFQs was to include the portion size of each food item (semi-quantitative FFQs) [29]. To assess the intake of calcium, phosphorus, vitamin D and protein from the diet, the the 24-h recall method was repeated twice. One weekday and one weekend day was included, and an average intake of 2 days. Dietary supplementation was also included in the interview. Questions included the amount and frequency of dairy product consumption and also the number of meals consumed per day. In this study, quantitative (g/person/day) and qualitative dietary data were collected for the three months preceding the survey. For the dietary interview, the 'Photo album of products and foods in different sizes' developed and published by the Institute of Food and Nutrition was used, as recommended [30]. Calcium intake (mg/day), protein intake (g/person/day), vitamin D intake (µg/day) and phosphorus intake (mg/day) were calculated in a computer programme for nutrition analysis (Diet 6.0).

### Physical activity and sedentary behaviour

Physical activity (PA) and sedentary time was assessed in a face-to-face interview with parents and boys conducted by an experienced interviewer using the International Physical Activity Questionnaire – Short Form (IPAQ-SF). In accordance with the IPAQ-SF methodology we assessed total PA level by collecting information on the number of days and the duration of vigorous-intensity PA, moderate-intensity PA and walking, as well as the duration of sitting for week-days in the preceding seven days [31]. This estimates the time spent sitting (screen time, computer games, school lesson) and in moderate to vigorous activities on weekdays. Average daily time spent in sitting (SIT time in hours per day and %time day) or moderate plus vigorous activity (MVPA in minutes per day and %time day) was calculated based on the estimates (Binkley and Specker 2016). The levels of PA (sufficient vs. insufficient) were adopted using the WHO Guidelines on Physical Activity and Sedentary Behavior [32].

### Statistical analysis

All calculations were performed using STATISTICA software (v.13.3, StatSoft, USA). In order to determine the significance of differences between the values of particular variables for boys with sufficient PA and insufficient PA a Student’s t test for independent variables was applied. Effect size was calculated using Cohen’s d = 2 t / (df^1/2), (small effect: < 0.5; medium effect: 0.5–0.8; large effect: > 0.8), [33]. The ANCOVA was applied in order to find relationships between bone parameters (BMD, BMC, Z-score, % age matched) and weight, moderate to vigorous PA, sitting and eating habits in young boys and level of PA (as qualitative predictor). ANOVA was used to evaluate the significance of differences in mean BMD and mean BMC in the context of PA and SIT time category. In order to determine the relationships between BMD and BMC and particular predictor variables, the multiple forward stepwise regression model was applied. The following levels of significance were used in the analyses: *p < 0.05; ** *p* < 0.01; *** *p* < 0.001 (*p*: *p*-value).

## Results

### Biometric, somatic and bones characteristics

The basic characteristics of the two groups (with sufficient and insufficient PA) of biometric, somatic, bones parameters and the significance of differences and effect sizes calculated using Cohen’s d are presented in Table 1. In somatic and body composition variables the groups differed significantly in 2 of 4 analysed parameters. The boys with insufficient PA had significantly higher weight, 4.7 kg, (medium effect d = 0.691) and height, 3.9 cm, (medium effect d = 0.672). In bone variables, the groups differed significantly in all 10 analysed parameters. Significantly lower (*p* < 0.001) all bone parameters in two part of forearm (large effect d > 0.8) was observed in boys with insufficient PA compared to sufficient PA. In PA and sedentary behaviour variables, the groups differed significantly in all 4 analysed parameters. Significantly lower (*p* < 0.001) MVPA (min/day) on average by 78.8 min/day and MVPA (%) (large effect d > 0.8) was observed in boys with insufficient PA compared to sufficient PA. An inverse relationship was shown for SIT time, with highest significance (*p* < 0.001) noted in SIT time (h/day) by 2.9 h/day and SIT time (%) (large effect d > 0.8) with boys with insufficient PA compared to sufficient. In eating habit variables, the groups differed significantly in all 6 analysed parameters. Significantly lower (*p* < 0.001) number of meals (n/day), dairy products (n/day) calcium intake (mg/day), protein intake (g/person/day) and vitamin D intake (µg/day) (large effect d > 0.8) was observed in boys with insufficient PA compared to sufficient PA. An inverse relationship was shown for phosphorus intake (mg/day), with highest significance (*p* < 0.001) noted in phosphorus intake by 131.8 mg/day (large effect d > 0.8) in boys with insufficient PA compared to sufficient (Table 1).

**Table 1:** Biometric, somatic and bones characteristics

**Variables**	**All** **(*n* = 115)**	**Sufficient PA (*n* = 51)**	**Insufficient PA (*n* = 64)**	***p*-value**	**Cohen’s d**
		**Mean ± SD**			
**Age (years)**	16.05 ± 1.02	15.9 ± 1.1	16.2 ± 0.9	0.093	0.300
**Somatic and body composition**					
**Weight (kg)**	67.5 ± 7.5	64.9 ± 5.3	69.6 ± 8,3	0.000**	0.691
**Height (cm)**	172.0 ± 6.3	169.8 ± 5.0	173.7 ± 6.6	0.000**	0.672
**BMI (kg/m**^**2**^**)**	22.8 ± 2.4	22.5 ± 1.6	23.1 ± 2.9	0.199	0.267
**Fat (%)**	14.9 ± 2.9	14.5 ± 1.9	15.2 ± 3.5	0.156	0.259
**Bone**					
**BMD dis (g/cm**^**2**^**)**	0.439 ± 0.117	0.502 ± 0.101	0.389 ± 0.104	0.000**	1.102
**BMC dis (g)**	1.724 ± 0.548	2.006 ± 0.381	1.500 ± 0.560	0.000**	1.075
**T-score dis**	0.085 ± 1.490	1.216 ± 1.180	-0.817 ± 1.024	0.000**	1.845
**Z-score dis**	0.218 ± 1.297	1.170 ± 1.074	-0.540 ± 0.902	0.000**	1.731
**% age matched dis**	98.9 ± 19.8	112.8 ± 16.8	87.9 ± 14.5	0.000**	1.591
**BMD prox (g/cm**^**2**^**)**	0.787 ± 0.206	0.909 ± 0.151	0.690 ± 0.192	0.000**	1.277
**BMC prox (g)**	2.243 ± 0.673	2.659 ± 0.574	1.911 ± 0.553	0.000**	1.327
**T-score prox**	-0.963 ± 1.472	0.224 ± 0.909	-1.910 ± 1.102	0.000**	2.122
**Z-score prox**	-0.576 ± 1.361	0.669 ± 0.938	-1.568 ± 0.633	0.000**	2.848
**% age matched prox**	85.8 ± 13.1	96.9 ± 9.1	77.0 ± 8.3	0.000**	2.287
**PA and sedentary behaviour**					
**MVPA (min/day)**	53.5 ± 49.5	97.4 ± 42.9	18.6 ± 13.7	0.000**	2.784
**MVPA (%)**	3.7 ± 3.4	6.8 ± 2.9	1.3 ± 0.9	0.000**	2.895
**SIT time (h/day)**	6.2 ± 1.7	4.5 ± 0.6	7.4 ± 1.1	0.000**	3.412
**SIT time (%)**	25.6 ± 7.1	18.9 ± 2.7	31.0 ± 2.9	0.000**	4.321
**Eating habits**					
**Number of meals (n/day)**	3.9 ± 1.0	4.5 ± 0.7	3.5 ± 0.9	0.000**	1.250
**Dairy products (n/day)**	1.7 ± 1.5	2.3 ± 1.3	1.1 ± 1.4	0.000**	0.889
**Calcium intake (mg/day)**	371.5 ± 129.7	431.8 ± 93.0	323.5 ± 135.2	0.000**	0.949
**Protein intake (g/person/day)**	56.9 ± 34.9	62.3 ± 8.4	46.9 ± 10.2	0.000**	1.656
**Vitamin D intake (µg/day)**	2.8 ± 0.8	3.44 ± 0.82	2.32 ± 0.43	0.000**	1.792
**Phosphorus intake (mg/day)**	1210.4 ± 171.1	1137.1 ± 109.5	1268.9 ± 189.7	0.000**	0.881

*Legend to tab.1*: *BMI* body mass index, *BMD* bone mineral density, *BMC* bone mineral content, *dis* distal, *prox* proximal, *PA* physical activity level, *MVPA* moderate to vigorous physical activity, *p p* –value; level of statistical significance *p < 0.05 and **p < 0.01; d- effect sizes calculated using Cohen’s formula

### Association between sedentary behaviour, eating habits, physical activity, and bone parameters

Analyses of the relationships of the characteristics studied (weight, MVPA, sitting and eating habits, PA as a continuous variable) with individual parameters of bone mineralization status separately for dis and prox segments are presented in Table 2. Of all the variables analysed, the strongest relationships were noted in BMD in the distal part for MVPA (min/day), SIT time (h/day), dairy products (n/day), calcium intake (mg/day), protein intake (g/person/day), vitamin D intake (µg/day), phosphorus intake (mg/day) and PA (level). However, SIT time proved to be the most important predictive variable of BMD in the distal part (*F* = 8.62; *p* = 0.001). The analysed set of features explained 61% of the variance in this parameter. The strongest relationships with BMD in the prox part were found for SIT time (*F* = 5.36; *p* = 0.023) and quantities of consumed dairy products per day (*F* = 5.99; *p* = 0.016). The analysed set of features explained 47% of the variance in this parameter, analogous analyses were performed for the BMC. The strongest relationships with BMC dis and prox were found for quantities of consumed dairy products per day, calcium, protein and phosphorus intake. However, dairy product intake proved to be the most important predictor variable of BMC; dis (*F* = 18.98; *p* = 0.001) and BMC prox (*F* = 13.66; *p* < 0.001). The analysed set of features explained 63% of the variance in theses parameters. This study also analysed the relationships of weight, physical activity, sitting and eating habits with Z-score and %age matched. The strongest relationships with Z-score in the dis region were observed for MVPA and eating habits (number of meals, intake of dairy products per day, protein and vitamin D intake). For Z-score for the prox region was most strongly influenced by SIT time and PA level. However, vitamin D intake proved to be the most important predictive variable of Z-score dis (*F* = 38.04; *p* < 0.001) and PA level for Z-score prox (*F* = 25.17; *p* < 0.001). The analysed set of features explained 70–79% of the variance in these parameters. The strongest relationships with % age matched dis were found for number of meals, protein and vitamin D intake. For % age matched prox was most strongly influenced by calcium intake and PA level. However, vitamin D intake proved to be the most important predictor variable of % age matched in dis (*F* = 21.54; *p* < 0.001) and PA level for % age matched prox (*F* = 20.82; *p* < 0.001), the analysed set of features explained 60–72% of the variance in this parameters. Relationships between bone parameters and weight, PA, sitting and eating habits in young boys (results of ANCOVA, F test, p) are shown Table 2.

**Table 2:** Relationships between bone parameters and weight, PA, sitting and eating habits in young boys

	**BMD dis**	**BMD prox**	**BMC dis**	**BMC prox**
	*F(p)*	*F(p)*	*F(p)*	*F(p)*
Weight (kg)	0.76 (0.386)	0.47 (0.494)	0.70 (0.406)	1.12 (0.293)
MVPA (min/day)	6.53 (0.012)**	2.82 (0.096)	2.69 (0.103)	0.15 (0.702)
SIT time (h/day)	8.62 (0.001)**	5.36 (0.023)*	2.15 (0.146)	2.20 (0.141)
Number of meals (n/day)	0.87 (0.352)	0.34 (0.563)	0.02 (0.885)	0.37 (0.547)
Dairy products (n/day)	5.14 (0.012)*	5.99 (0.016)*	18.9 (0.000)**	13.7 (0.000)**
Calcium intake (mg/day)	3.86 (0.052)*	2.84 (0.095)	2.71 (0.057)	0.63 (0.430)
Protein intake (g/person/day)	6.02 (0.015)*	0.01 (0.939)	13.6 (0.000)**	13.5 (0.000)**
Vitamin D intake (µg/day)	6.55 (0.012)**	0.03 (0.872)	0.62 (0.433)	3.14 (0.079)
Phosphorus intake (mg/day)	3.94 (0.050)*	0.45 (0.503)	4.55 (0.035)*	6.91 (0.009)**
PA (level)	5.59 (0.019)*	0.05 (0.826)	0.35 (0.554)	0.15 (0.702)
**F(p)** **Rˆ2 adj**	18.71 (0.000) 0.61	11.09 (0.000) 0.47	20.06 (0.000) 0.63	20.20 (0.000) 0.63
	Z-score dis	Z-score prox	% age matched dis	% age matched prox
	*F(p)*	*F(p)*	*F(p)*	*F(p)*
Weight (kg)	0.07 (0.792)	0.31 (0.579)	1.29 (0.258)	0.50 (0.479)
MVPA (min/day)	4.19 (0.043)*	1.85 (0.177)	1.02 (0.315)	2.12 (0.149)
SIT time (h/day)	2.15 (0.146)	9.09 (0.003)**	0.83 (0.364)	0.20 (0.655)
Number of meals (n/day)	19.26 (0.000)**	0.15 (0.698)	8.48 (0.004)**	0.19 (0.665)
Dairy products (n/day)	3.64 (0.053)*	1.10 (0.298)	1.68 (0.198)	0.30 (0.585)
Calcium intake (mg/day)	0.98 (0.324)	2.98 (0.087)	0.03 (0.872)	5.50 (0.021)*
Protein intake (g/person/day)	13.89 (0.000)**	0.15 (0.698)	16.6 (0.000)**	0.13 (0.718)
Vitamin D intake (µg/day)	38.04 (0.000)**	0.96 (0.329)	21.5 (0.000)**	2.41 (0.123)
Phosphorus intake (mg/day)	0.05 (0.828)	1.10 (0.298)	2.66 (0.106)	0.88 (0.351)
PA (level)	1.02 (0.314)	25.2 (0.000)**	0.01 (0.985)	20.8 (0.000)**
**F(p)** **Rˆ2 adj**	43.29 (0.000) 0.79	28.19 (0.000) 0.70	30.96 (0.000) 0.72	18.02 (0.000) 0.60

*Legend to tab.2*: *BMD* bone mineral density, *BMC* bone mineral content, *dis* distal, *prox* proximal, *PA* physical activity, *MVPA* moderate to vigorous physical activity, *F* Ronald A. Fisher’s test, *p p*-value; level of statistical significance *p < 0.05 and **p < 0.01, *Rˆ2 adj* the adjusted R-squared values of determination

Relationships between BMD, BMC in the distal and proximal part of the forearm and PA, sit time and eating parameters were evaluated using the multiple forward stepwise regression (Table 3). The presented model explained 48–67% (adjusted *R*2 = 0.48–0.67; *p* < 0.001) of the variance in bone parameters. The validity of the predictors in the model was compared using Standardized β. The predictor of interactions of three variables: protein intake (g/person/day), vitamin D intake (µg/day) and phosphorus intake (mg/day) was significant for BMD dis (adjusted R2 0.59; *p* < 0.001). The predictor of interactions of two variables: SIT time (h/day) and dairy products (n/day) was significant for BMD prox (adjusted *R*2 = 0.48; *p* < 0.001). Furthermore, the predictor of interactions dairy products (n/day), protein intake (g/person/day) and phosphorus intake (mg/day) was significant for BMC prox and dis (adjusted *R*2 = 0.63–0.67; *p* < 0.001). Relationships between BMD and BMC in the distal and proximal part of the forearm and physical activity, sit time and eating parameters (multiple forward stepwise regression) shows Table 3.

**Table 3:** Relationships between BMD and BMC—multiple forward stepwise regression

**Bone parameters**	**Predictor**	**Standardized β**	**Adjusted R**^**2**^	**F (p)**
**BMD dis**	MVPA (min/day)	0.126	0.59	24.6 (< 0.001)
	SIT time (h/day)	-0.141		
	Dairy products (n/day)	0.171		
	Calcium intake (mg/day)	0.151		
	Protein intake (g/person/day)	0.225		
	Vitamin D intake (µg/day)	0.209		
	Phosphorus intake (mg/day)	0.148		
**BMD prox**	MVPA (min/day)	0.192	0.48	16.07 (< 0.001)
	SIT time (h/day)	-0.275		
	Dairy products (n/day)	0.249		
	Calcium intake (mg/day)	0.144		
	Protein intake (g/person/day)	0.068		
	Vitamin D intake (µg/day)	0.009		
	Phosphorus intake (mg/day)	0.059		
**BMC dis**	MVPA (min/day)	0.111	0.63	28.85 (< 0.001)
	SIT time (h/day)	-0.072		
	Dairy products (n/day)	0.375		
	Calcium intake (mg/day)	0.124		
	Protein intake (g/person/day)	0.367		
	Vitamin D intake (µg/day)	0.051		
	Phosphorus intake (mg/day)	0.140		
**BMC prox**	MVPA (min/day)	0.099	0.67	34.94 (< 0.001)
	SIT time (h/day)	-0.117		
	Dairy products (n/day)	0.313		
	Calcium intake (mg/day)	0.012		
	Protein intake (g/person/day)	0.367		
	Vitamin D intake (µg/day)	0.144		
	Phosphorus intake (mg/day)	0.173		

*Legend to tab.3*: *BMD* bone mineral density, *BMC* bone mineral content, *dis* in distal part of forearm, *prox* in proximal part of forearm, *MVPA* moderate to vigorous physical activity

**Fig. 1:**
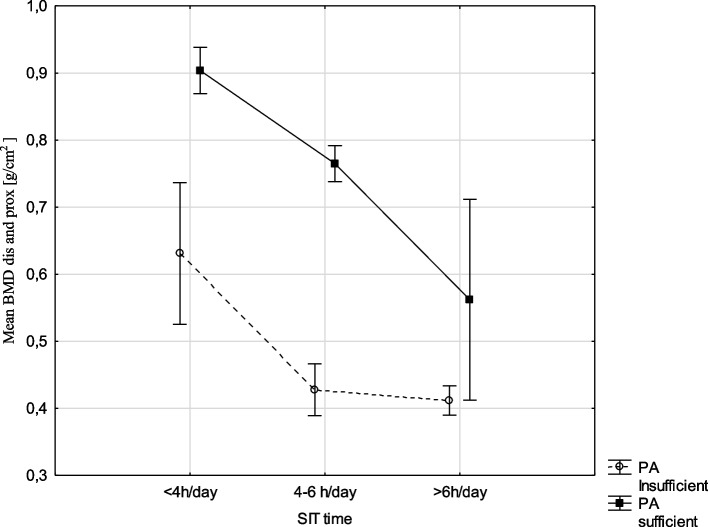
Relationship between mean BMD (g/cm^2^) among level of physical activity and SIT time (h/day),(results two ways ANOVA analysis, F(2, 109) = 3,0537, *p* = ,05124), vertical lines -0.95 CI—confidence intervals. Abbreviation: BMD, bone mineral density; PA, physical activity

**Fig. 2:**
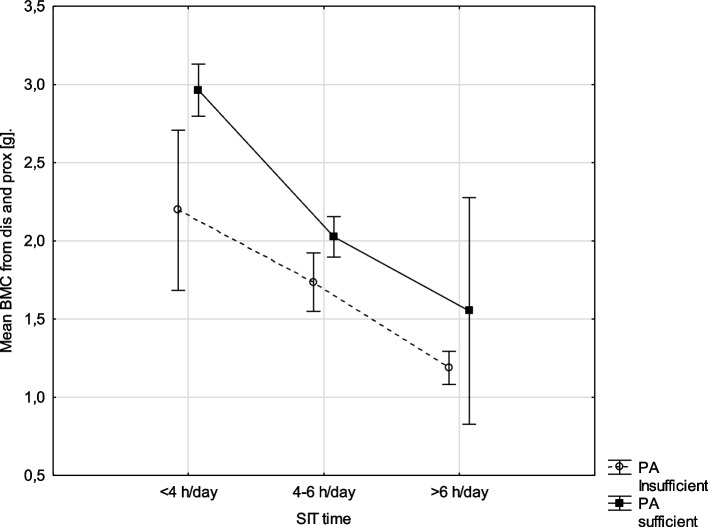
Relationship between mean BMC (g) among level of physical activity and SIT time (h/day) (results twoways ANOVA analysis, F(2, 109)=1,3160, *p*=,27244), vertical lines -0.95 CI - confidence intervals. Abbreviation: BMC, bone mineral content; PA, physical activity

Figures 1 and 2 presents a graphical representation of the results of a two-factor ANOVA analysis of variance. Mean BMD dis and prox was dependent on the level of PA and was significantly highest in sufficiently active boys with the smallest SIT time (< 4 h/day). BMD values decreased with increasing SIT time in both groups of boys (Fig. 1).

An analogous analysis was performed for the mean BMC (dis and prox), regardless of level PA, boys with smallest SIT time had the most advantageous values of Mean BMC (Fig. 2).

## Discussion

The condition of bone tissue in the adolescent years is important for bone health in adulthood and senior years. Underestimated bone mineralization increases the risk of osteoporosis with studies pointing to a number of factors affecting bone mineralization at different skeletal locations. In this study, BMD and BMC were found to be related to sedentary behaviour, eating habits and level of PA with different strengths at two points on the forearm. In this study the boys with insufficient PA had significantly higher weight, (+ 4.7 kg), and significantly lower all bone parameters in two parts of the forearm, significantly lower MVPA (min/day), significantly highest SIT time (h/day) by 2.9 h/day compared to sufficient PA group. This study also analysed eating habits and in particular the intake of dietary components important for bone health. Significantly lower number of meals (n/day), dairy products (n/day) calcium intake (mg/day), protein intake (g/person/day) and vitamin D intake (µg/day) but significantly highest phosphorus intake by 131.8 mg/day was observed in boys with insufficient compared to sufficient PA. In this study, time of MVPA and optimal eating habits especially adequate intake of calcium, protein, vitamin D and phosphorus affect the mineralization of forearm bones in boys.

Previous studies have shown that bone mineral density in a young population is determined by many factors [5, 8, 12, 14]. Previous studies, with the participation of various age groups, have observed the influence of different levels of PA on bone however the results are not consistent. Kennedy et al. [34] observed MVPA to be positively associated with hip BMC, and spine bone area in children nearly 7 years old. The results of Binkley et al. [12] showed no significant association of MVPA with better bone status. Kopiczko et al. [22] observed that the type and level of PA proved to be an important determinant of forearm bone parameters in boys. In this study significant differences were observed in BMD and BMC between groups of boys who are involved in track and field sports, classified as forms of activity with high osteogenic index (OI), and groups of not training and also training in the water. It was noted that swimming and physically inactive boys had the weakest bone status [22]. Tebar et al. analysed the relationship between PA levels and regional BMD in children and adolescents aged 6–14 years,oys with low PA levels had lower BMD Z-score for arms and legs compared to highly physically active youths [35].

In this present study of all the variables analysed, the strongest relationships with BMD in distal part were found for MVPA (min/day), SIT time (h/day), dairy products (n/day), calcium intake (mg/day), protein intake (g/person/day), vitamin D intake (µg/day), phosphorus intake (mg/day) and PA (level). However, SIT time proved to be the most important predictive variable of BMD in the distal part. The strongest relationships with BMD in prox part were found for SIT time and quantities consumed dairy products per day. The strongest relationships with BMC dis and prox were found for quantities of consumed dairy products per day, calcium, protein and phosphorus intake. However, dairy product intake proved to be the most important predictor variable of BMC dis and BMC prox. In addition, present study showed that in both of the group with sufficient PA and insufficient PA, the highest BMD and BMC values were in boys with the lowest SIT time < 4 h/day.

In research of boys and girls, aged 9.5 ± 1.5 years, Christofaro et al. [13] compared BMD according to different domains of sedentary behavior (SB), findingthat subjects with low total SB had higher BMD in legs when compared to subjects with high total SB. The authors found that high levels of sedentary behaviour resulted in significantly lower whole-body bone mineral density compared to groups with low levels of sedentariness [13]. Shao et al. [36] in a study about determinants of BMD in Chinese adolescents, demonstrated an inverse relationship between longer sit time for playing video games and BMD from different body regions. The authors noted the possible mechanisms, and found that the shorter the time spent in passive, sedentary positions, the more time remains for PA [36]. Our results confirm those of other studies, that sedentary behavior and especially long time spent in sedentary positions can influence the reduction of BMD in various skeletal locations in children and adolescents.

Bone status also depends on eating habits from an early age, in the present study the strongest relationships with BMD in distal part were found for dairy products (n/day), calcium intake (mg/day), protein intake (g/person/day), vitamin D intake (µg/day), phosphorus intake (mg/day) and the strongest relationships with BMD in prox part were found for quantities of consumed dairy products per day. The strongest relationships with BMC dis and prox were found for quantities of consumed dairy products per day and calcium, protein and phosphorus intake, the results of present study are consistent with those of other authors.

In a number of studies, dairy products or those fortified with Ca and/or vitamin D have had a positive impact on BMD and/or BMC [37, 38]. Dairy products, dairy products, eggs are often recommended especially in the diet of children and adolescents as a good source of calcium and protein, important factors of milk and dairy products are probiotics and prebiotics, which may modulate bone turnover. Probiotics and prebiotics significantly affect the microbial balance of the intestines and this, in turn, determine the proper course of processes affecting the condition of bone tissue. The intestinal microflora influences osteoclasts while also acting on the intestinal absorption of calcium [39]. As emphasized by Weaver, milk and products prepared from milk are a very good source of bioavailable calcium from the diet [40]. Research results indicate that in particular fermented milk products have a positive effect on bone growth and mineralization, and are also important in the prevention of osteoporosis [41]. Among children and adolescents with diets low in dairy products an increased risk of fractures of bones has been reported, this is similar to adults or older individuals who follow a diet devoid of dairy products [42]. Nutritional experts emphasize the important role of milk and dairy products in the prevention of osteoporosis [43] with several studies indicating that good bone status is dependent on higher dietary protein intake, provided that the calcium supply is sufficient. Dairy products are a valuable source of these two nutrients [44]. During the course of bone formation the rate at which peak bone mass is built, is an important determinant of bone health in the life stages following childhood. At the same time, it is crucial to recognize the importance of vitamin D levels from the prenatal period to adolescence [45, 46]. Two meta-analyses [11, 46] examining the relationship of dairy consumption to BMD or BMC in children and adolescents demonstrated the significance of the beneficial effects of dairy products on bone parameters in children. However, four showed no effect.

The relevance of dairy produce for bone status is still a matter of scientific debate, more research is required to understand the role of dairy products within the context of bone health-promoting diets in specific ethnicities and different aged populations. The determination ofone mineral density is multifactorial. It is important to continue research in this area in order to fully understand the mechanisms of influence of lifestyle, especially physical activity, eating habits and sedentary behavior (SB). 

### Study strengths and limitations

The research was conducted by a highly qualified team. The design of this study was prepared in accordance with the recommendations of bone tissue research, using proven research tools. The strength of the present study is that a reliable research methodology was used and adapted to the age of the studied boys. The densitometric examination was performed by a specialist qualified in radiological diagnostic methods. This project also pays significant attention to the use of well-chosen data analysis methods to eliminate indirect effects.

This study had some limitations. It is acknowledged that there is a sample size of just over 100 boys and this may be considered insufficient, however, in order to deepen the analysis of the determinants of BMD in the young population future cohort studies should be conducted. Future studies should also consider assessing BMD at several skeletal locations in addition to the forearm alone.

### Implications

The findings suggest that there is important correlation between bone health in adolescent boys and lifestyle factors such as sedentary behaviour, eating habits and level of physical activity. Further research is needed to clarify whether improving eating habits and increasing physical activity, while reducing the amount of time spent sitting, may be a key factor in the prevention of low bone density and thus osteoporosis in adulthood. The results of this study can be used as a practical implication for new programs to counter the sedentary lifestyle and its impact on the health of the growing population.

## Conclusions

In this study, physical activity level especially time in moderate to vigorous physical activity (MVPA), sitting (SIT) and eating habits proved to be an important determinants of forearm bone parameters in young Polish boys. We observed significantly lower bone mineral density and bone mineral content in boys with insufficient level of physical activity compared to active boys with sufficient PA. High MVPA and optimal eating habits, especially adequate intake of important dietary components for bone health such as calcium, protein, vitamin D and phosphorus affect the mineralization of forearm bones. Paying more attention to sufficient length of moderate to vigorous physical activity (MVPA) in young population can help offset the effects of high SIT time. In this study, maintaining sufficient levels of physical activity according to WHO recommendations was associated in the study group with better eating habits and less sedentarism.

## Data Availability

The selected range of data and materials is available after direct contact with the correspondence author.

## Abbreviations

BMC: Bone mineral content
BMD: Bone mineral density
BR: Bone remodeling
DIS: Distal part of forearm
DXA: Dual-energy X-ray absorptiometry
IPAQ-SF: International Physical Activity Questionnaire – Short Form
MVPA: Vigorous and moderate intensity physical activity
PA: Physical activity
PBM: Peak bone mass
PROX: Proximal part of forearm
SIT TIME: Daily time spent in sitting
WHO: World Health Organization
